# Probiotics, prebiotics, and postbiotics in health and disease

**DOI:** 10.1002/mco2.420

**Published:** 2023-11-04

**Authors:** Jing Ji, Weilin Jin, Shuang‐Jiang Liu, Zuoyi Jiao, Xiangkai Li

**Affiliations:** ^1^ MOE Key Laboratory of Cell Activities and Stress Adaptations School of Life Sciences Lanzhou University Lanzhou Gansu China; ^2^ Institute of Cancer Neuroscience Medical Frontier Innovation Research Center The First Hospital of Lanzhou University The First Clinical Medical College of Lanzhou University Lanzhou Gansu China; ^3^ State Key Laboratory of Microbial Resources Institute of Microbiology Chinese Academy of Sciences Beijing China; ^4^ Cuiying Biomedical Research Center The Second Hospital of Lanzhou University Lanzhou Gansu China

**Keywords:** clinical trials, gut microbiota, postbiotics, prebiotics, probiotics

## Abstract

The gut microbiota and its homeostasis play a crucial role in human health. However, for some diseases related to the gut microbiota, current traditional medicines can only relieve symptoms, and it is difficult to solve the root causes or even cause side effects like disturbances in the gut microbiota. Increasing clinical studies and evidences have demonstrated that probiotics, prebiotics, and postbiotics can prevent and treat various diseases, but currently they can only be used as dietary supplements rather than medicines, which restricts the application of probiotics in the field of medicine. Here, this review analyzes the importance of gut microbiota in human health and the current problems of traditional medicines, and systematically summarizes the effectiveness and mechanisms of probiotics, prebiotics, and postbiotics in maintaining health and treating diseases based on animal models and clinical trials. And based on current research outcomes and development trends in this field, the challenges and prospects of their clinical application in maintaining health, alleviating and treating diseases are analyzed. It is hoped to promote the application of probiotics, prebiotics, and postbiotics in disease treatment and open up new frontiers in probiotic research.

## Graphical abstract

Extensive research and clinical evidence have demonstrated the mechanisms and effectiveness of probiotics, prebiotics, and postbiotics in restoring gut microbiota homeostasis and treating a variety of diseases. Prebiotics can promote the growth of beneficial intestinal bacteria, while probiotics produce postbiotics through “addition” to regulate the gut microbiota, and “subtraction” to remove harmful metabolites and exogenous substances to reduce their impact on the body, thereby alleviating or treating diseases. Probiotics, prebiotics, and postbiotics may represent the next generation of medicines, with the potential to revolutionize the way we treat and manage disease.

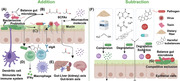

## 1 INTRODUCTION

The human gut microbiota is an intricate ecosystem comprising trillions of microorganisms, including bacteria, viruses, fungi, and other microbial entities. This dense and diverse microbiome has coevolved with humans, shaping and being shaped by our physiology, diet, and lifestyle.^1^ This vast microbial community plays a multifaceted role in our gut, and its homeostasis has emerged as an indispensable aspect of human health. Recent scientific explorations have revealed that gut microbiota homeostasis is critical for regulating intestinal inflammation, maintaining human metabolic homeostasis, and maturation and regulation of the immune system.^2^ However, disruptions in gut microbiota homeostasis, known as dysbiosis, may contribute to the development of a variety of diseases.^3^ For instance, inflammatory bowel disease (IBD), obesity, type 2 diabetes, and even distant systemic diseases, including cardiovascular diseases, certain cancers, and neurodegenerative disorders.^4^, ^5^ Furthermore, research on the gut–brain axis has further highlighted that dysbiosis may modulate neurochemical pathways that exacerbate mental health problems such as depression and anxiety.^6^ In summation, the equilibrium of our gut microbiota is a linchpin in maintaining health and staving off disease. For the related diseases caused by the imbalance of gut microbiota homeostasis, the selection of effective treatment methods and interventions to restore and maintain gut microbiota homeostasis is the key to achieving overall health and corresponding disease treatment.

Synthetic medicines have become the linchpin of contemporary therapeutic strategies, providing therapeutic avenues for a myriad of ailments.^7^ Leveraging the nuances of molecular biology and chemistry, synthetic medicines can target aberrant pathways with remarkable specificity, exhibiting a high degree of efficacy and predictability in treating a plethora of ailments.^8^ With their capacity for tailored design, synthetic medicines have reshaped the therapeutic landscape, offering solutions where traditional treatments had limited impact.^9^ However, as our understanding of human health evolves, it becomes increasingly evident that diseases are seldom an outcome of isolated molecular malfunctions.^10^, ^11^ In particular, diseases caused by disturbances in the gut microbiota.^12^ This revelation presents a conundrum in the context of synthetic medicine treatments. While these medicines exhibit proficiency in targeting specific molecules or pathways, they often fall short and produce certain side effects when the disease etiology involves the intricate and dynamic balance of gut microbiota.^13^ Diseases rooted in gut microbiota disturbances often require holistic, multifaceted interventions. While synthetic medicines might address specific symptoms, they may not adequately restore microbial balance, thus merely offering symptomatic relief without addressing the root cause.^14^ Furthermore, synthetic medicines, particularly broad‐spectrum antibiotics that attack nonspecifically, potentially instigating or exacerbating health issues associated with gut dysbiosis.^15^ Thus, while synthetic medicines represent a monumental stride in disease treatment, their interaction with the gut microbiota poses significant challenges necessitates a more holistic approach.

With the growing evidence on the pivotal role of the gut microbiota in human health and disease, the importance of probiotics, prebiotics, and postbiotics in the medical field has also been amplified due to their ability to promote health and treat disease through modulation of the gut microbiota.^16^, ^17^, ^18^ Probiotics are live microorganisms that provide health benefits when consumed in adequate amounts^19^; prebiotics are nondigestible substances (typically dietary fibers) that promote the growth and activity of beneficial gut bacteria^20^; and postbiotics are the active substances produced by probiotics during their growth, and contribute to gut health.^21^ Probiotics are live beneficial microbes, prebiotics are food for these microbes, and postbiotics are the beneficial compounds produced by probiotics. Each plays a distinct role in the symbiotic relationship with the human host and significantly contributes to the body's overall homeostasis.^22^ Probiotics are often consumed in fermented foods or dietary supplements and have been studied for their potential to improve gut health, immune function, and more.^17^ Prebiotics beneficially affect the host by selectively stimulating the growth and activity of one or several types of bacteria in the colon.^23^ They essentially serve as “food” for the beneficial bacteria in the gut, helping to increase their numbers and promote a healthier microbiome.^20^ Postbiotics represent the newest field of study among the three. They are the health‐conferring substances that probiotics produce when they metabolize dietary components, signifying a return to the roots of probiotic research.^21^, ^24^ In recent years, probiotics, prebiotics, and postbiotics have made breakthroughs in the treatment of various human systemic diseases.^25^ As a feasible strategy, probiotics, prebiotics, and postbiotics have shown potential as medicine‐alternative or complementary therapies to promote health and slow disease progression.

Given the ability of probiotics, prebiotics, and postbiotics to modulate the gut microbiota, maintain health, and treat disease.^26^, ^27^ Understanding the effects and mechanisms of probiotics, prebiotics, and postbiotics and promoting their clinical application represents an essential frontier in biomedical research. However, since prebiotics, postbiotics, and even probiotics are only classified as health products rather than medicines, public acceptance and regulatory rules also limit their application in the medical field.^20^, ^21^, ^28^ Therefore, this review first affirms the importance of gut microbiota in human health, reviews the development of medicines, and summarizes the limitations of traditional medicines in targeting gut microbiota‐related diseases. On this basis, it summarizes the current knowledge of probiotics, prebiotics, and postbiotics, their advantages in health and disease treatment, exemplifies their recent breakthroughs in animal experiments and clinical trials, and summarizes the main roles mechanisms to demonstrate the effectiveness and feasibility of probiotics, prebiotics, and postbiotics in maintaining human health and treating diseases. Simultaneously, the current challenges faced by probiotics, prebiotics, and postbiotics in clinical application promotion are analyzed, and future development is prospected. It is hoped that current regulatory rules can be gradually changed, thereby accelerating the research and clinical application of probiotics, prebiotics, and postbiotics and promoting public acceptance of this new treatment method.

## 2 IMPORTANCE OF GUT MICROBIOTA IN HEALTH AND DISEASE

The gut microbiota, often referred to as our “second genome,” is an intricate assembly of microorganisms residing within the human gastrointestinal tract. This microbiome contains more than 100 trillion microbes, outnumbering our own cells.^29^ In terms of composition, the gut microbiota is dominated by bacteria, with more than 1000 known species of bacteria, 90% of which are *Firmicutes* and *Bacteroidetes*.^30^ *Firmicutes* include *Clostridium*, *Lactobacillus*, and *Ruminococcus* and other genera, members of this group ferment dietary fiber into short‐chain fatty acids (SCFAs) such as butyrate, which serves as a primary energy source for the cells lining our colon and plays a pivotal role in regulating inflammation.^31^ *Bacteroidetes*, particularly the genera *Bacteroides* and *Prevotella*, are adept at breaking down complex carbohydrates, thereby releasing essential nutrients that our bodies would otherwise find inaccessible.^32^ Other bacterial phyla, while less abundant, holds its significance. *Bifidobacteria* within the *Actinobacteria* phylum are a representative genus celebrated for their probiotic attributes, influencing gut health by strengthening the mucosal barrier and warding off potential pathogens.^33^ Another intriguing member is the *Proteobacteria* phylum. Though it includes some opportunistic pathogens, such as certain *Escherichia* and *Salmonella* species, it also houses beneficial strains like the *Dendroctonus rhizophagus* can metabolize uric acid in insects to maintain nitrogen balance, elucidating the duality of the gut environment.^34^ The *Verrucomicrobia* phylum, albeit in smaller quantities, has gained attention for *Akkermansia muciniphila*. This bacterium interacts with the mucus layer of the intestine, playing roles in both its maintenance and overall gut health.^35^ Less explored phyla such as *Fusobacteria* and *Cyanobacteria*, though minor in terms of abundance, add to the diversity of the gut microbiota and potentially play roles yet to be unraveled.^36^

**FIGURE 1:**
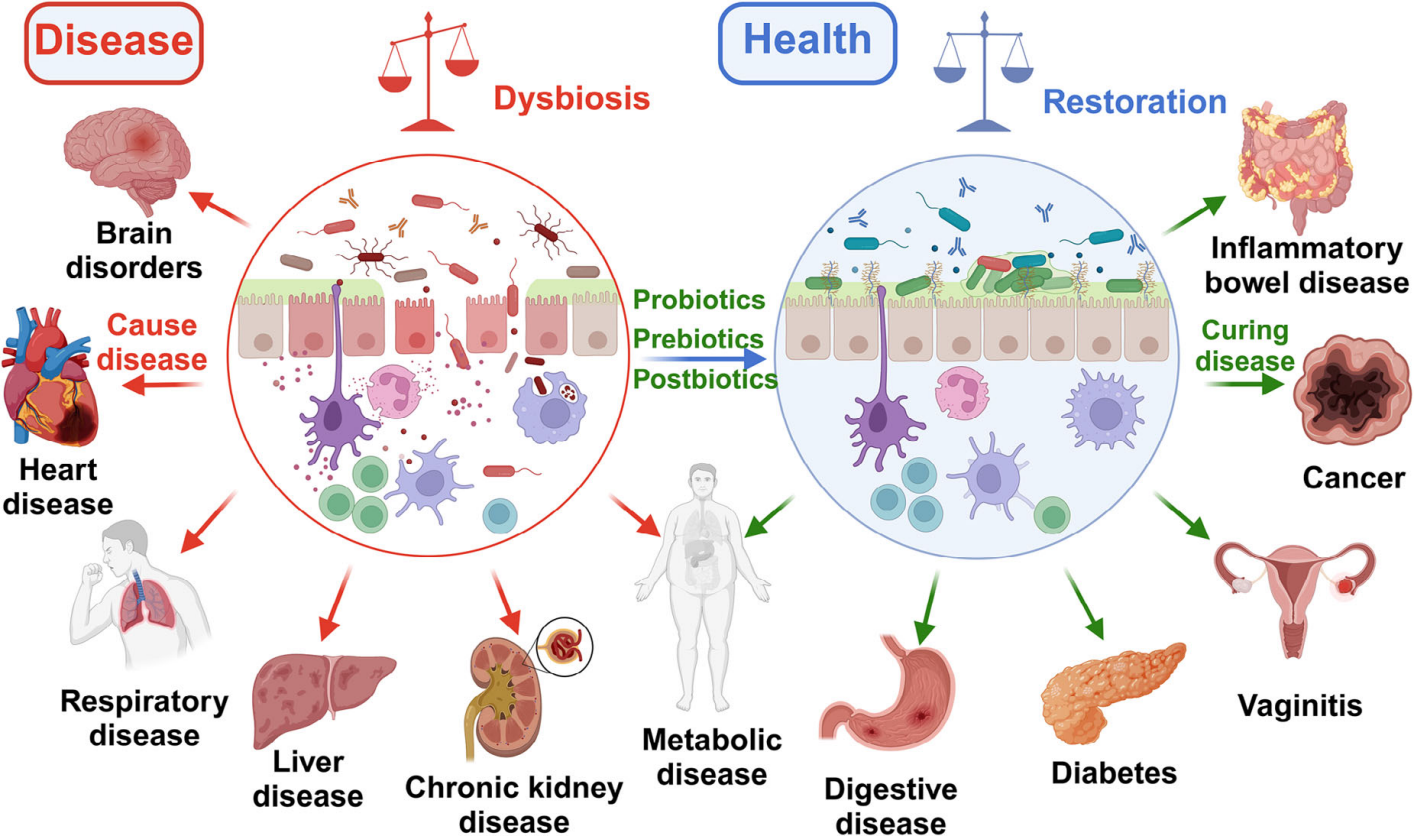
Diseases associated with gut microbiota dysbiosis, and probiotics, prebiotics, and postbiotics to alleviate or treat disease by restoring gut microbiota homeostasis.

Most of the bacteria in the intestinal tract are benign or neutral, but some of the conditionally pathogenic bacteria may cause serious threats to human health once they multiply uncontrollably.^37^ Dysbiosis of gut microbiota homeostasis can lead to dysfunction and disease, including cardiovascular disease, bacterial infection, kidney disease, obesity, cancer, IBD, and so on (Figure 1).^12^, ^38^ Within the *Enterobacteriaceae* family of the gut, although many strains of *E. coli* are harmless and are a part of the normal gut microbiota, the overgrowth of pathogenic strains, such as *E. coli O157:H7*, can lead to severe gastrointestinal diseases and even systemic complications.^39^ Another member, *Salmonella*, can cause food poisoning, manifesting as severe diarrhea and fever.^40^ These harmful bacteria not only damage the intestinal lining but also release toxins that cause severe damage to the human system. *Clostridium difficile* is an infamous pathogen within the *Firmicutes* phylum. While *C. difficile* can exist harmlessly in the gut, its overgrowth, caused by the disruption of gut microbiota by broad‐spectrum antibiotics, can lead to debilitating diarrhea and, in severe cases, life‐threatening colitis.^41^ The colonization of *Helicobacter pylori* in the gastrointestinal tract can lead to ulcers and increase the risk of stomach cancer.^42^ Some strains within the *Clostridiales* order, especially *Fusobacterium nucleatum*, have been found to be associated with colorectal cancer.^43^ An increase in the abundance of conditionally pathogenic bacteria exacerbates disease development, and the onset of disease and the evolution of the gut microbial community usually influence each other bidirectionally.

Increasing evidence suggests that the bidirectional communication between the gut microbial community and the host may facilitate the development of diseases, and abnormalities in the abundance of certain gut microbes during disease also hint at the impact of the gut microbiota on health and disease.^44^, ^45^ In a 4‐year study of 16 women with lupus from diverse ethnic backgrounds, five experienced a significant proliferation of the gut bacterium *Ruminococcus blautia gnavus* concurrent with disease flare‐ups.^46^ In gastrointestinal diseases, the decrease of butyrate‐producing bacteria like *Prevotella copri* and *Roseburia* could influence the growth of colon cells and reduced mucus production.^47^ Meanwhile, an increase in mucolytic bacteria like *Ruminococcus* species, virulent *Escherichia coli*, and *Bacteroides fragili*s might result in mucosal damage and exacerbated bacterial invasion.^48^ In metabolic‐related diseases, it is commonly observed that obese individuals have increased abundance of *Firmicutes*, *Bacteroidetes*, *Clostridia*, and *Lactobacillus*, while the abundance of *Bacteroides*, *Faecalibacterium prausnitzii*, *A. muciniphila*, *Lactobacillus paracasei*, and *Lactobacillus plantarum* decreased.^49^ Type 2 diabetes is positively correlated with *Clostridium*, *Ruminococcus*, and *Blautia* but negatively with *Bacteroides*, *Bifidobacterium*, *Pseudobacteroides*, and *Parabacteroides*.^50^ In central nervous system‐related diseases like Parkinson's disease (PD) and Alzheimer's disease (AD), a decrease in *Prevotella*, *Pseudobacteroide*s, and *Faecalibacterium* is observed, while *Bifidobacterium* and *Akkermansia* levels increase.^51^, ^52^ Although current research presents some varied results, each bacterial group in the gut plays specific roles. Essentially, the composition of the gut microbial community is a dynamic mosaic that continuously changes based on diet, environment, and host factors, and these shifts impact human health and disease progression.

The composition of the gut microbiota, while resilient, is easily influenced by various factors.^53^ A deeper understanding of these determinants is crucial for therapeutic interventions aiming to correct diseases associated with ecological imbalances. At birth, whether through vaginal delivery or cesarean section, the foundation for an infant's gut microbiota is established.^54^ Babies born via vaginal delivery inherit microbial features similar to their mother's vaginal tract, while those born via cesarean section exhibit bacteria characteristics associated with the skin.^55^ One of the primary influencers of microbial composition is diet. A diet rich in fiber can promote the growth of beneficial bacteria such as *Bifidobacterium* and *Lactobacillus*, producing SCFAs beneficial for gut health.^56^ In contrast, diets high in fat, protein, or sugar might promote the proliferation of potentially harmful bacteria associated with inflammation, such as *Bacteroidetes* and *Biliophiles*.^57^ Environmental factors, such as exposure to pathogens, pollutants, and even varying climates, can cause shifts in gut microbial balance.^58^ Lifestyle choices, including exercise frequency, alcohol consumption, and stress levels, are also associated with specific microbial features.^59^ Age, genetics, and underlying health conditions further influence and shape the gut microbiota.^60^ Moreover, the use of medications, especially broad‐spectrum antibiotics, while widely employed to treat infections and imbalances caused by opportunistic pathogens, has been shown in numerous studies to induce temporary and even long‐term changes in the composition, diversity, and functionality of the gut microbiota.^61^ The gut microbiota is closely related to human health and diseases. How to maintain or restore the corresponding gut microbiota homeostasis and function while targetedly treating ecological imbalances‐related diseases has become a current research hotspot.

## 3 PROBIOTICS, PREBIOTICS, AND POSTBIOTICS THERAPY EMERGES AS ALTERNATIVE MEANS OF DISEASE TREATMENT

### 3.1 Current issues and limitations of traditional medicines

Improving human health and treating diseases mainly depend on medicines, which are substances designed to prevent, diagnose, and treat diseases, as well as regulate physiological functions and promote overall health.^62^ They can be categorized into natural products derived from plants, animals, or microorganisms; artificial and chemically synthesized compounds; and biologics.^63^ Due to nearly two centuries of technological advances in the fields of chemistry, biology, and pharmacology, many types of medicines have been developed that have revolutionized medical treatment.^64^, ^65^, ^66^ However, in recent years, with the increasing difficulties of screening, medicine development also has faced several bottlenecks hindering progress. High failure rates lead to wasted resources and extended development timelines, as a significant proportion of medicine candidates fail during preclinical and clinical trials, primarily due to lack of efficacy or safety concerns.^67^ The lengthy development process, which typically takes 10−15 years and involves multiple stages such as target identification, lead optimization, preclinical testing, and clinical trials, is further complicated by regulatory approval processes.^68^ Increased difficulty in drug screening, high failure rates and lengthy development processes drive high costs, with an average research and development (R&D) cost of more than $2.8 billion per approved medicine.^69^ The total investment in new medicine R&D has been increasing year by year, while the number of approvals has not increased, and it has become more and more difficult to develop new traditional medicines. At the same time, although currently developed medicines have many benefits, they also have some limitations, which may impact their effectiveness and patient outcomes.

Many medicines cause mild to severe side effects, impacting patients quality of life and treatment adherence.^70^ Some Chinese herbal extracts used in antiobesity treatment may also cause drug‐induced liver injury.^71^ Furthermore, some medicines may not work for all patients due to genetic variations, disease heterogeneity, or medicine resistance, leading to suboptimal treatment outcomes.^72^ Patients on multiple medications may experience interactions that reduce medicine effectiveness or cause unexpected side effects.^73^ Nonadherence to prescribed treatment regimens due to complexity, forgetfulness, or concerns about side effects can also reduce medicine effectiveness and cause complications. Some medicines have a narrow therapeutic window, making optimal dosing difficult without risking toxicity or subtherapeutic levels.^74^ Recent research indicate that most human diseases were associated with changes in the composition of gut microbiota.^49^, ^75^, ^76^ However, conventional medicines treatments are often struggle to effectively target the gut microbiota, alleviate the diseases related to the gut microbiota and difficult to fundamentally solve the cause of some metabolic diseases, and even cause undesirable side effects such as flora disturbance.^77^, ^78^

The interplay between medicines and the composition of gut microbiota challenges prior concepts of medicine specificity and reveals broader physiological impacts. Numerous clinical studies have long demonstrated the effects of antibiotics on the gut microbiota, where they might reduce beneficial bacteria, leading to a decrease in microbial diversity, causing everything from short‐term gastrointestinal disturbances to increased long‐term risks of conditions like IBD and obesity.^79^, ^80^ Emerging research suggests that the influence of medicines on gut microbiota is not solely limited to antibiotics. Vich Vila et al.^81^ explored the effects of commonly used medicines on gut microbial composition and metabolic functions, finding that 19 out of 41 medicines were associated with microbial features, with proton pump inhibitors, metformin, and laxatives displaying the strongest correlations with microbiota. Proton pump inhibitors are used to suppress stomach acid production and treat conditions such as peptic ulcers and gastroesophageal reflux, but they result in a significant decline in gut microbial diversity and an increased abundance of potentially pathogenic genera like *Enterococcus*, *Streptococcus*, and *Staphylococcus*.^82^ Chemotherapy medicines also have a particularly pronounced effect on the gut microbiota, decreasing the relative abundance of most genera while promoting the growth of pathogenic strains.^83^ They might also damage the intestines, leading to inflammation and diarrhea.^84^ Considering the limitations of current traditional medicine in the treatment of gut microbiota‐related diseases and the adverse effects of traditional medicines on gut microbiota homeostasis, coupled with the demand for disease treatment and the development of biomedical technology, there is an urgent need to develop and promote new treatment methods.^85^ Fortunately, probiotics, prebiotics, and postbiotics have received more and more attention because they can regulate the gut microbiota, which seems to provide a breakthrough to solve the limitations of traditional medicines.

### 3.2 Definitions and overview of probiotics, prebiotics, and postbiotics

**FIGURE 2:**
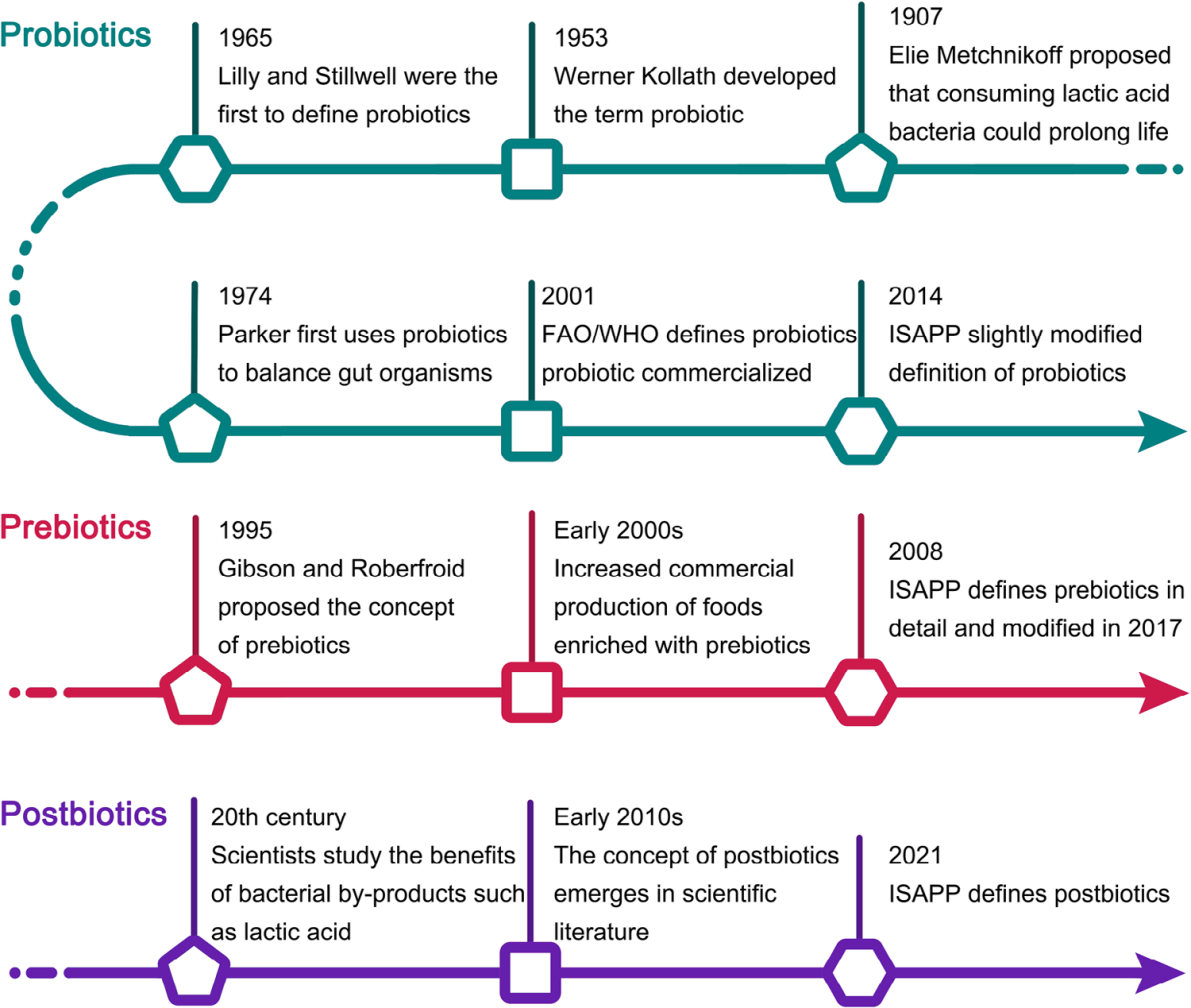
History of the development of probiotics, prebiotics, and postbiotics. FAO, Food and Agriculture Organization of the United Nations; WHO, World Health Organization; ISAPP, International Scientific Association for Probiotics and Prebiotics.

The term probiotic, derived from the Greek words “pro” and “bios” meaning “for life.” It was originally discovered by Elie Metchnikoff that eating lactic acid bacteria can prolong life.^86^ Lilly and Stillwell^87^ first defined probiotics in 1965, used to describe substances produced by one microorganism that stimulates the growth of another. Today, the most widely accepted definition is that issued by the Food and Agriculture Organization of the United Nations (FAO) and the World Health Organization (WHO) in 2001, which defines probiotics as “live microorganisms which, when administered in adequate amounts, confer a health benefit on the host.”^19^ A century has passed from the discovery of beneficial microbes to the definition of probiotics (Figure 2). There are many types of probiotics that come from different families of bacteria and yeasts. Some of the most commonly recognized genera of bacteria used as probiotics include *Lactobacillus*, *Bifidobacterium*, *Enterococcus*, and *Streptococcus*, as well as the yeast *Saccharomyces cerevisiae*.^88^ Each genus contains several species, and within each species, there are many strains. The health effects of probiotics are generally considered to be strain specific.^89^ The precise mechanisms of probiotics are complex and depend on the specific strains. However, some common mechanisms have been identified. Probiotics can alter the gut microbiota composition, compete with pathogens for nutrients and binding sites on the intestinal wall, enhance the intestinal barrier function, and modulate the immune system.^90^ They can also produce antimicrobial substances and other metabolites that can directly or indirectly influence host health.^91^ Additionally, they can influence the nervous system of the host, communicating through the gut–brain axis.^92^ Through the above‐mentioned abilities of probiotics to modulate the composition and/or activity of the gut microbiota, they can help prevent or alleviate various diseases such as IBD, irritable bowel syndrome (IBS), and metabolic syndrome.^93^

Prebiotics were first defined in 1995 as “non‐digestible food ingredients that beneficially affect the host by selectively stimulating the growth and/or activity of one or a limited number of bacteria in the colon.”^94^ Over the years, the definition has been refined, and today prebiotics are recognized as dietary components that are selectively utilized by host beneficial microorganisms conferring a health benefit, according to the International Scientific Association for Probiotics and Prebiotics (ISAPP).^20^ Most prebiotic compounds are carbohydrates with a variety of molecular structures that occur naturally in the human diet. Common types of prebiotics include inulin, fructooligosaccharides (FOS), galactooligosaccharides (GOS), and lactulose. They can be found in various foods such as whole grains, onions, garlic, and bananas.^89^ Prebiotics work primarily by providing a food source for beneficial bacteria in the gut. They are nondigestible by human enzymes, so they reach the colon intact where they are fermented by the gut bacteria.^95^ This fermentation process results in the production of SCFAs, including acetate, propionate, and butyrate.^96^ Prebiotics selectively stimulate the growth and activity of beneficial gut bacteria such as *Bifidobacteria* and *Lactobacilli*, reduce the abundance of pathogenic bacteria, and thus help to maintain a balanced gut microbiota.^97^ Simultaneously produced metabolites can provide energy to the cells lining the colon, regulate the immune system, enhance gut barrier function, and even influence brain function through the gut–brain axis.^98^

The definition of postbiotics was released by ISAPP in 2021, which refers to preparation of inanimate microorganisms and/or their components that confers a health benefit on the host.^21^ They include a wide range of components, such as inactivated microbial cells, cell wall components, functional proteins, peptides, SCFAs, polyamines, vitamins, bacteriocins, and other bioactive metabolites.^24^ The exact composition of the postbiotics, which are produced by probiotic metabolism, depends on the probiotic strain used, the growth conditions, and the substrates available for fermentation.^99^ Postbiotics exert their beneficial effects via several mechanisms, similar to probiotics and prebiotics. They can modulate the gut microbiota composition by inhibiting the growth of harmful bacteria and enhancing the function of beneficial bacteria, enhance the gut barrier function, exhibit anti‐inflammatory and antioxidant properties, modulate the immune response.^100^ In addition, some prebiotics, like SCFAs, can also act as signaling molecules that influence host metabolism. They can communicate with the host cells through the microbial‐associated molecular patterns recognized by the host's pattern recognition receptors.^101^ Because they are nonviable and do not replicate in the gut, postbiotics present a safer alternative to probiotics for immunocompromised individuals or critically ill patients.^102^ Although there are significant differences among probiotics, prebiotics, and postbiotics, both prebiotics and postbiotics have a close relationship with probiotics (Table 1). They either promote the growth and metabolism of probiotics or result from the metabolism of probiotics, ultimately promoting the homeostasis of the intestinal microbiota and helping to treat or alleviate host diseases.

**TABLE 1:** A comparison of the differences between probiotics, prebiotics, and postbiotics.

	Probiotics	Prebiotics	Postbiotics
Definition	Live microorganisms that, when administered in adequate amounts confer a health benefit on the host.^19^	Nondigestible food ingredients that beneficially stimulate the growth and/or activity of gut bacteria.^20^	A preparation of inanimate microorganisms and/or their components that confers a health benefit on the target host.^21^
Source	Bacteria or yeast, often from fermented foods like yogurt. Examples: *Lactobacillus*, *Bifidobacterium*.	Typically dietary fibers or other carbohydrates. Examples include inulin, FOS, and GOS.	Typically metabolic byproducts from probiotic bacteria. Can be extracted and administered without live bacteria.
Function	Can colonize the gut, enhancing its microbial balance. They can produce postbiotics.^90^	Provide food for beneficial bacteria, promoting their growth and activity.^97^	May not directly affect microbiota composition but exert beneficial effects on host health.^100^
Stability	Sensitive to environmental conditions like temperature and stomach acid.^103^	Generally stable and not affected by temperature or stomach acid.	Stable; not sensitive to temperature, stomach acid, or digestive enzymes.
Safety	Can cause infections in immunocompromised individuals.^104^	Overconsumption can lead to gastrointestinal discomfort.^105^	Generally safe, but the effects of large amounts are not well‐known.^100^
Examples	Yogurt, kefir, fermented foods, certain supplements.	Foods like garlic, onions, asparagus, and whole grains.	SCFAs like butyrate, certain bacterial cell components, or peptides.

Abbreviations: FOS, fructooligosaccharides; GOS, galactooligosaccharides; SCFAs, short‐chain fatty acids.

### 3.3 Probiotics, prebiotics, and postbiotics for maintaining health and treating disease

In recent years, research on the gut microbiota and its role in health and disease has gradually become a hot topic.^76^ The gut microbiota plays a key role in the development and maintenance of the human immune system's good health.^106^ Based on the limitations of current traditional medicine and the treatment of gut microbiota‐related diseases, the demand for disease treatment, and the development of biomedical technology, it is urgent to promote new treatment methods.^85^ As a feasible strategy, probiotics, prebiotics, and postbiotics have shown potential as alternative or complementary therapies to promote health and slow disease progression by restoring the balance of the gut microbiota.^16^, ^17^ In recent years, they have made breakthroughs in treating diseases of the digestive system, immune system, cardiovascular system, and nervous system (Figure 1).^107^ Localized treatment effectively targets gastrointestinal disorders like IBS, IBD, antibiotic‐associated diarrhea, and even colorectal cancer.^70^ Their impact on the gut–brain axis suggests potential applications in neuropsychiatric treatment, including anxiety and depression.^108^, ^109^

Probiotics, usually derived from human gut microbiota or foods, along with prebiotics and postbiotics, have been demonstrated as safe through long‐term consumption and extensive research.^110^ They generally exhibit fewer risks of medication interactions compared to traditional synthetic or chemically derived pharmaceutical treatments, making them a safer option for patients on multiple medications.^73^ In comparison with some conventional medicines that can lead to tolerance, dependence, or other adverse reactions, they are more suitable for long‐term use and are friendlier to vulnerable groups.^111^ Moreover, the mechanisms by which probiotics and the postbiotics produced by their metabolism can prevent or treat various diseases have essentially been proven through extensive clinical trials.^23^ Given their abundant resources, the development of functional probiotics, prebiotics, and postbiotics is less challenging, offering a natural treatment option that appeals to those seeking alternatives to synthetic medications.

In recent years, the United States Food and Drug Administration has proposed and defined the concept of “live biotherapeutics” such as some bacteria that are not vaccines but are capable of preventing, treating, or curing certain diseases.^112^ Probiotics, which are safer than common microorganisms, can be promoted by prebiotics to increase their abundance in the host gut, subsequently producing various postbiotics that can combat a range of diseases. However, due to varying regulations, management rules across countries, and levels of public acceptance, probiotics, prebiotics, and postbiotics have traditionally been classified as dietary supplements rather than medicine, which has limited their research and application in disease treatment.^28^ Given the effectiveness and unique advantages of probiotics and their metabolites in treating a variety of diseases, and considering the definitions of medicines and “live biotherapeutics,”^62^ we believe it is necessary to systematically emphasize the role of probiotics, prebiotics, and postbiotics in preventing, alleviating, and treating disease. According to the current in‐depth R&D trend in the field of probiotics, prebiotics and metabolically generated postbiotics, they may represent the next generation of medicines, boasting immense potential for development and application. This could revolutionize our approach to disease treatment and management.

## 4 EFFECTIVENESS AND MECHANISMS OF PROBIOTICS AND POSTBIOTICS IN TREATING DISEASES

### 4.1 Evidences for the effectiveness of probiotics and postbiotics derived from probiotics in treating disease in animal models

In recent years, an increasing number of studies have reported the application of probiotics and postbiotics produced by their metabolism in the treatment of various animal disease models, the efficacy of probiotics, with its varying functions in treating corresponding diseases, has demonstrated the potential of probiotics, prebiotics, and postbiotics as medicine. Based on the fact that postbiotics are produced by the growth or metabolism of probiotics and are the main substances for the functions of probiotics, here, we combine them together for discussion and analysis.^21^ In gastrointestinal diseases, probiotics such as *Bifidobacterium longum* and *Limosilactobacillus fermentum* KBL374, have been shown to alleviate symptoms of IBS and dextran sulfate sodium (DSS)‐induced IBD in mice by modulating the gut microbiota, immune response, and improving intestinal mucosal barrier function.^113^, ^114^ In cancer prevention and treatment, Bender *et al*. found that *Lactobacillus reuteri* can stimulate cytotoxic CD8^+^ T cells by secreting postbiotics indole‐3‐aldehyde (I3A).^115^ When mice were fed a tryptophan‐rich diet and metabolized by *L. reuteri* into I3A, it enhanced the effectiveness of immune checkpoint inhibitor (ICI) treatment, suppressed melanoma size, and prolonged survival.

The *A. muciniphila* (ATCC BAA‐835^T^), isolated from the human gut by van Passel et al.,^116^ showed the ability to inhibit the progression of ovarian cancer in mice by increasing acetate levels to activate T cells.^117^ Additionally, research on *A. muciniphila* in other mouse disease models has shown that specific metabolites or membrane proteins of *A. muciniphila* can be linked to host cell types or receptors, targeting the potential of various diseases through the liver–brain–gut axis, such as obesity, diabetes, metabolic syndrome, nonalcoholic steatohepatitis, and even neurodegenerative diseases.^118^, ^119^ For infection prevention, *Bacillus subtilis* ZK3814 has been reported to eliminate *S. aureus* by secreting postbiotic fengycin, which inhibits *S. aureus* quorum sensing.^120^ The VSL#3 compound probiotic capsule, developed by VSL Pharmaceuticals, can not only treat IBS by increasing the secretion of SCFAs, but also prevent respiratory syncytial virus infection in mice by regulating the microbiome‐alveolar‐macrophage axis to stimulate IFN‐β production.^121^, ^122^ The treatment of the above‐mentioned diseases is linked to the postbiotics secreted by probiotics, showing the effectiveness and importance of probiotics in treating and alleviating diseases.

Utilizing the unique metabolic capabilities of probiotics to target the degradation of endogenous metabolites or exogenous pollutants has also shown effectiveness in the treatment of corresponding animal disease models.^123^ Engineered probiotics, such as *EcN‐Cbh*, have been developed for dynamically modulating intestinal metabolism; *EcN‐Cbh* can deconjugate taurocholate into cholate, that limited the germination of endospores and the growth of vegetative cells of *Clostridioides difficile in vitro*, and further significantly inhibited *C. difficile* infection in model mice.^124^ Hyperuricemia is a condition characterized by elevated levels of uric acid in the blood, which can lead to gout and kidney stones.^125^ *L. fermentum* JL‐3, a strain isolated from “Jiangshui” was found to ameliorate hyperuricemia by degrading uric acid, and improved renal function in hyperuricemic mice.^126^ Hyperoxaluria caused by endogenously synthesized and exogenously ingested oxalates, is also a major contributor to kidney stone formation.^127^ The oxalate‐degrading probiotic *Oxalobacter formigenes* is isolated from the human gut and relies solely on oxalate for growth.^128^ *O. formigenes* can effectively colonize the intestine and efficiently degrade soluble oxalate through two key enzymes, formyl‐CoA‐transferase and oxalyl‐CoA‐decarboxylase, and reduce the risk of kidney stones by breaking down dietary and endogenous oxalates.^129^, ^130^

In the detoxification of exogenous pollutants, *Pediococcus acidilactici* strain BT36, isolated from Tibet plateau yogurt, enhances the reduction of Cr(VI) by the gut microbiota in mice while promoting their excretion from the body, mitigating the toxic effects of heavy metals.^131^ Probiotics such as *Lactiplantibacillus plantarum* P9, *L. plantarum* 20261, and *Pediococcus pentosaceus* ATCC 43200 have been shown to degrade organophosphorus pesticides in vitro.^132^, ^133^, ^134^ *Bacillus cereus* GW‐01 can also alleviate the accumulation and harmful effects of β‐cypermethrin in mice through bioadsorption.^135^ Foodborne toxic substances such as aflatoxin and zearalenone can also be degraded by probiotics.^136^, ^137^ Compared with conventional medicine treatments, the direct degradation of toxic substances by probiotics is a more effective and uniquely advantageous bioremediation strategy. Probiotics, as a promising therapeutic approach, have demonstrated their effectiveness in the treatment of various animal disease models (Table 2).

**TABLE 2:** Report on probiotics treating diseases in animal models (2018‐2023).

Target disease	Model	Probiotic	Treatment effect	Mechanism	Refs
IBS	Male Sprague–Dawley rats, water chronic exposure	*Bifidobacterium longum*	Improved defecation habits, visceral ↓hypersensitivity, ↑mucosal repair	↑Lysozyme production, ↑stem niche factors WNT3A and TGF‐β	^114^
IBD	Female C57BL/6N mice, DSS induced	*Limosilactobacillus fermentum* KBL374	↑Colon length, ↓inflammatory cytokines, ↑body weight, ↓leukocyte infiltration	Regulating immune responses, altering gut microbiota, ↑gut barrier function	^113^
CRC	Male C57BL/6 ‐APC^Min/+^, DSS induced	*L. plantarum* YYC‐3	↓Occurrence of colon tumors and mucosal damage, ↓inflammatory cytokines, ↓VEGF‐MMP2/9 signaling pathway	Immunomodulation, altered gut microbiota, secreted metabolites	^138^, ^139^
Melanoma	C57BL/6 WT mice, breast cancer cells induced	*Lactobacillus reuteri*	↑ICI efficacy, promoting ICI response and patients survival	Secretes indole‐3‐aldehyde to stimulate CD8^+^ T cells	^140^
Infection	Female C57BL/6J mice, S. aureus and antibiotics induced	*Bacillus subtilis* ZK3814	Eliminates *S. aureus*, suppressed production of Agr‐regulated virulence factors	ZK3814 secretes fengycins inhibit quorum sensing	^120^
Autism spectrum disorders	Shank3 KO mice	*L. reuteri*	Modifies social and repetitive behaviors, ↓GABA receptor expression, ↑hypothalamic expression of oxytocin	Brain–gut axis	^141^
Depression	Male ICR mice, loperamide induced	Compound probiotics	Ameliorate depressive behaviors, ↓neuronal cell injury, ↓Bax and cleaved caspase‐3, ↑p‐AKT and Bcl‐2 levels	Activating the AKT signaling pathway	^142^
Alcoholic liver disease	Male C57BL/6 mice, alcohol induced	*Bifidobacterium breve* ATCC15700	↓Endotoxemia, maintained immune homeostasis, alleviated liver injury, ↑tight junction proteins	Promoted intestinal barrier function, regulate gut microbiota	^143^
Hyperuricemia	Male Kunming mice, oteracil potassium and UA‐induced	*Limosilactobacillus fermentum* JL‐3	↓Serum UA level (31.3%), ↓oxidative stress indicators	Degrade uric acid, regulate gut microbiota	^126^
Chromate poisoning	Female Kuming mice, Cr (VI) induced	*Pediococcus acidilactici strain* BT36	↓Chromate accumulation, ↓oxidative stress	Promotes chromium excretion, regulate gut microbiota	^131^
Kidney stones	Male Rattus novergicus, calcium oxalate induced	*L. casei* 01	Attenuates the development of renal calculi	Degrade and utilize oxalate	^144^
Toxins	Male ICR mice, aflatoxin B1 gavage	*L. plantarum* T3	↑Aflatoxin B1 excretion, ameliorate oxidative stress and immune imbalance	Removal aflatoxin B1 by cell adsorption, restore gut homeostasis	^145^
Diabetic	Male Kunming mice, glucose induced	Compound probiotics	↓Blood glucose, ↓SGLT‐1 and GLUT‐2 expression, ↓intestinal permeability	Probiotics competitively consume glucose	^146^

Abbreviations: AKT, protein kinase B;CRC, colorectal cancer; GLUT‐2, glucose transporter 2.; SGLT‐1, recombinant sodium/glucose cotransporter 1.

### 4.2 Breakthroughs of probiotics and postbiotics in clinical trials

**FIGURE 3:**
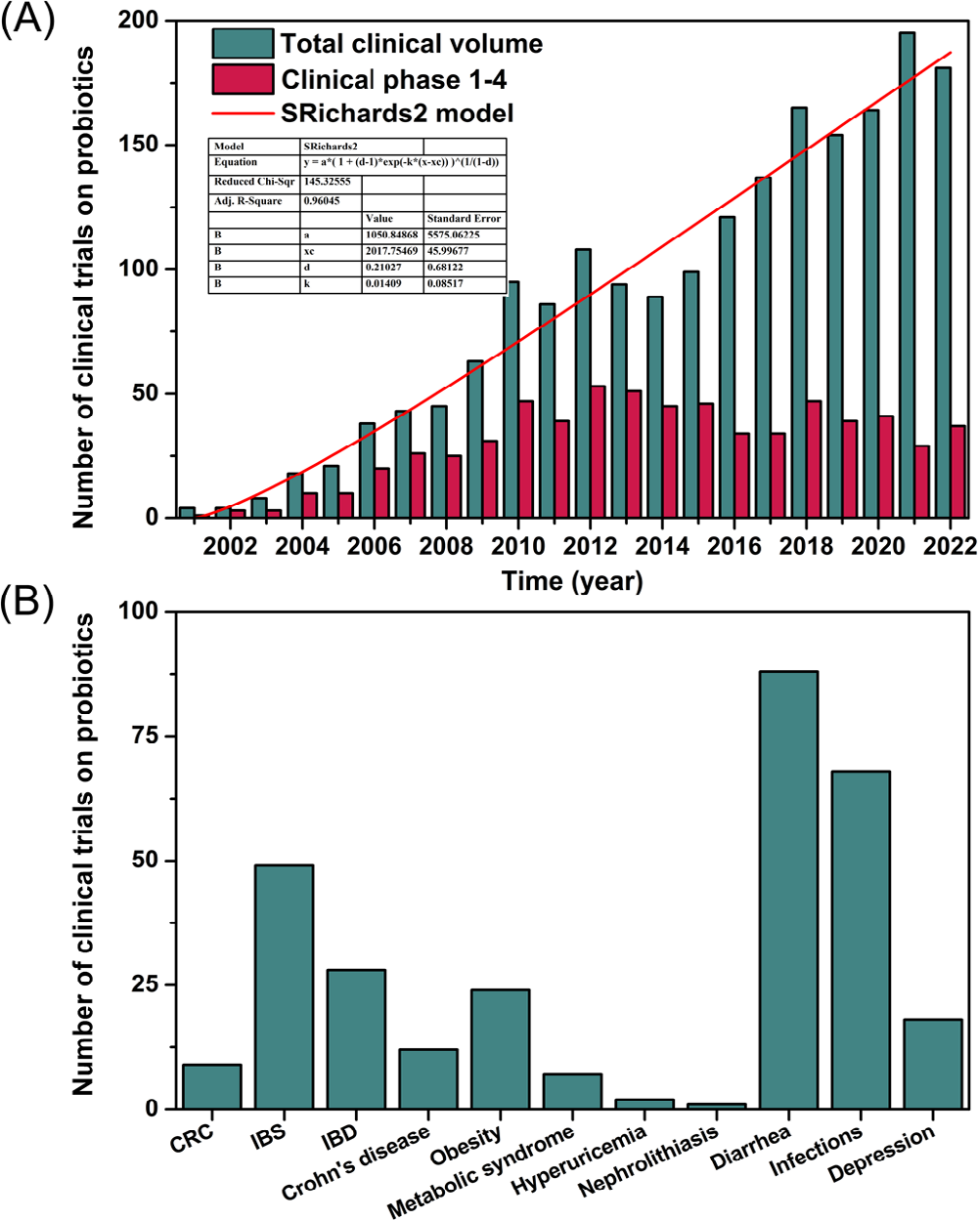
Trends in clinical trials of probiotics since the 21st century. (A) Annual number of clinical trials and phases 1−4 of clinical trials of probiotics registered in the ClinicalTrials.gov database, and the growth fitting model of the total number of clinical trials (2001–2022), (B) and the main ones that scientists are interested in disease type. CRC, colorectal cancer; IBS, irritable bowel syndrome; IBD, inflammatory bowel disease. Data from https://clinicaltrials.gov/.

Numerous preclinical studies have demonstrated the disease‐treatment mechanisms and effectiveness of probiotics and postbiotics, making them a focus point of clinical research in recent years due to their potential benefits and unique therapeutic advantages. According to the number of clinical trial registrations in the ClinicalTrials.gov database (https://clinicaltrials.gov/), probiotics‐related clinical research has grown rapidly since 2001 (Figure 3A). Based on growth model data fitting, the growth rate of probiotics clinical research is found to align with the Richards model, with an estimated 1000 studies per year projected for the future. Although the number of registered phase 1−4 clinical trials of probiotics has slowed down in recent years, it has remained at around 40 per year and is expected to increase in 2023. Furthermore, statistics on probiotics clinical research in some common diseases from 2001 to 2023 show that researchers have mainly focused on conditions like IBS, IBD, diarrhea, and infections (Figure 3B). This could be related to the proven primary therapeutic mechanisms of probiotics, such as regulating gut microbiota, modulating immune responses, and improving gastrointestinal function.

In recent years, probiotics and postbiotics have achieved breakthroughs in clinical research across various diseases. They complement each other and both play a key role in regulating the intestinal flora and maintaining human health. In the treatment of melanoma, *L. reuteri* can colonize in melanoma and promote CD8^+^ T cells production of interferon‐γ by releasing the tryptophan metabolite postbiotic I3A, thereby enhancing the efficacy and survival of ICIs in advanced melanoma patients (NCT02112032).^115^ Clinical research by Spencer et al.^147^ also confirmed the benefits of probiotic supplementation in immunotherapy for melanoma. Breakthrough results have also been achieved in the use of probiotics to combat infant malnutrition. A commercial U.S. donor‐derived strain of *B. longum* subspecies (EVC001), complexed with prebiotic human milk oligosaccharides, has been shown to increase the abundance of *Bifidobacteria* in the gut of infants with severe acute malnutrition. EVC001 completely degrades and utilizes human milk oligosaccharides through exo‐α‐sialidase NanH2 and α‐fucosidases to generate monosaccharides and SCFAs that maintain the balance of gut microbiota (NCT03666572).^97^, ^148^ Furthermore, together with the Indole‐3‐lactic acid produced by metabolizing tryptophan, it is beneficial in reducing intestinal inflammation and promoting weight gain in infants.^149^

Hyperuricemia is a typical metabolic disorder. In a randomized, double‐blind, controlled study of 120 hyperuricemia patients, it was proven that the probiotics *Limosilactobacillus fermentum* GR‐3 improves human hyperuricemia by degrading and promoting uric acid excretion, significantly reducing serum uric acid levels (26.2 ± 2.3%) (Chinese Clinical Trial Registry, ChiCTR2100053287).^150^ Exposure to heavy metals poses a threat to human health, by giving 152 occupational workers from the metal industry drinking yogurt containing *L. plantarum* GR‐1, the effectiveness of GR‐1 in reducing heavy metal levels and alleviating exposure toxicity by altering the gut microbiota and metabolome was demonstrated (ChiCTR2100053222).^151^ In addition, the synergistic treatment of probiotics and conventional therapy has also been carried out clinically.^152^ The coadministration of *Bifidobacterium animalis* subsp. *Lactis* Probio‐M8, benserazide, and dopamine agonists has improved patient sleep quality, alleviated anxiety and gastrointestinal symptoms, and enhanced the clinical efficacy of treating PD (ChiCTR1800016977).^153^

Based on clinical research on probiotics registered in the ClinicalTrials.gov database in the past five years, the Bio‐25 probiotics capsules by Ambrosia‐SupHerb company for treating IBD and IBS, PerioBalance® by SUNSTAR Suisse SA company as an adjunct treatment for peri‐implant mucositis or peri‐implantitis, *Bifidobacterium breve* strains BR03 and B632 for treating obesity, and Lacidofil^®^ for preventing flu incidence in the elderly have completed phase 4 clinical trials. In addition, the BIO‐25 probiotics capsules have already hit the market and become a well‐known probiotic product. Table 3 also lists the evidence from some representative clinical trials registered in the ClinicalTrials.gov database. With the growing attention to probiotics and postbiotics resources and advances in techniques among researchers, an increasing number of probiotics and postbiotics will be obtained and discovered.^16^, ^154^ Breakthroughs in clinical research on probiotics and their generated postbiotics lays the foundation for probiotics as medicines, while also holding enormous potential to transform the field of medicine.

**TABLE 3:** Evidence of probiotics in clinical trials (2018‐2023).

Target disease	Probiotic	Aim	Phase	NCT Number
CRC	*L. rhamnosus* TCELL‐1	Evaluation the Effectiveness of *L. Rhamnosus* TCELL‐1 Upon CRC	Phase 2	NCT05570942
IBS	Probiotic combination (BIO‐25)	Assess the clinical effects of the multispecies probiotic combination “BIO‐25″ in IBS‐D patients	Phase 4	NCT01667627
IBD	Probiotic formula capsule	Beneficial effects of probiotic adjuvant therapy in ulcerative colitis patients	Phase 2 Phase 3	NCT04223479
Mucositis	*L. reuteri* Prodentis (PerioBalance®)	Evaluation of the effect of *L. reuteri* Prodentis in the treatment of mucositis and periimplantitis	Phase 4	NCT03047291
Antibiotic‐associated diarrhea	*Bifidobacterium animalis subsp*. Lactis BB‐12	The role of probiotics in prevention of antibiotic‐associated diarrhea	Early Phase 1	NCT03755765
Type 2 diabetes mellitus	*Bifidobacterium animalis subsp. Lactis* (BPL‐1)	Evaluate the efficacy and safety of BPL‐1 treatment in adult patients with type 2 diabetes mellitus	Phase 2	NCT04191525
Metabolic syndrome	*L. helveticus* Rosell®−52, *B. longum* Rosell®−175	Assessing the effect of probiotics on metabolic syndrome	Phase 3	NCT04756544
Obesity	*B. breve* BR03 and B632	Evaluating the efficacy of probiotics in weight loss	Phase 4	NCT03261466
Acne vulgaris	Probiotic formula (Hi‐Flora)	Measure the efficacy of probiotics for treatment of acne vulgaris	Phase 3	NCT05629468
HIV infections	*L. Plantarum*, *S. Thermophiles*, *B. Bifidum* “Rillus^®^”	Discover Rillus^®^ improves gut inflammation in HIV patients.	Phase 2	NCT03568812
Influenza‐like illness (ILI)	*L. acidophilus* Rossel‐52, *L. rhamnosus* Rosell‐11	Asses the effect of probiotic on enhancing immune response to ILI and reducing incidence	Phase 4	NCT03695432
Helicobacter infection	*L. acidophilus* and *L. rhamnosus*	Evaluating efficacy of probiotics reduce bacterial load of H. pylori and modify gut microbiotia	Phase 2 Phase 3	NCT02725138
Social stress	*B.longum* 1714	Investigate if probiotics can improve response to social stress in healthy participants	Phase 2 Phase 3	NCT02793193
Depression	Formula probiotic	Investigated the effect of formula probiotic on depression	Phase 1	NCT04567147

All data in the Table 3 are from https://clinicaltrials.gov/.

### 4.3 The main mechanisms of probiotics and postbiotics to maintain health and treat disease

**FIGURE 4:**
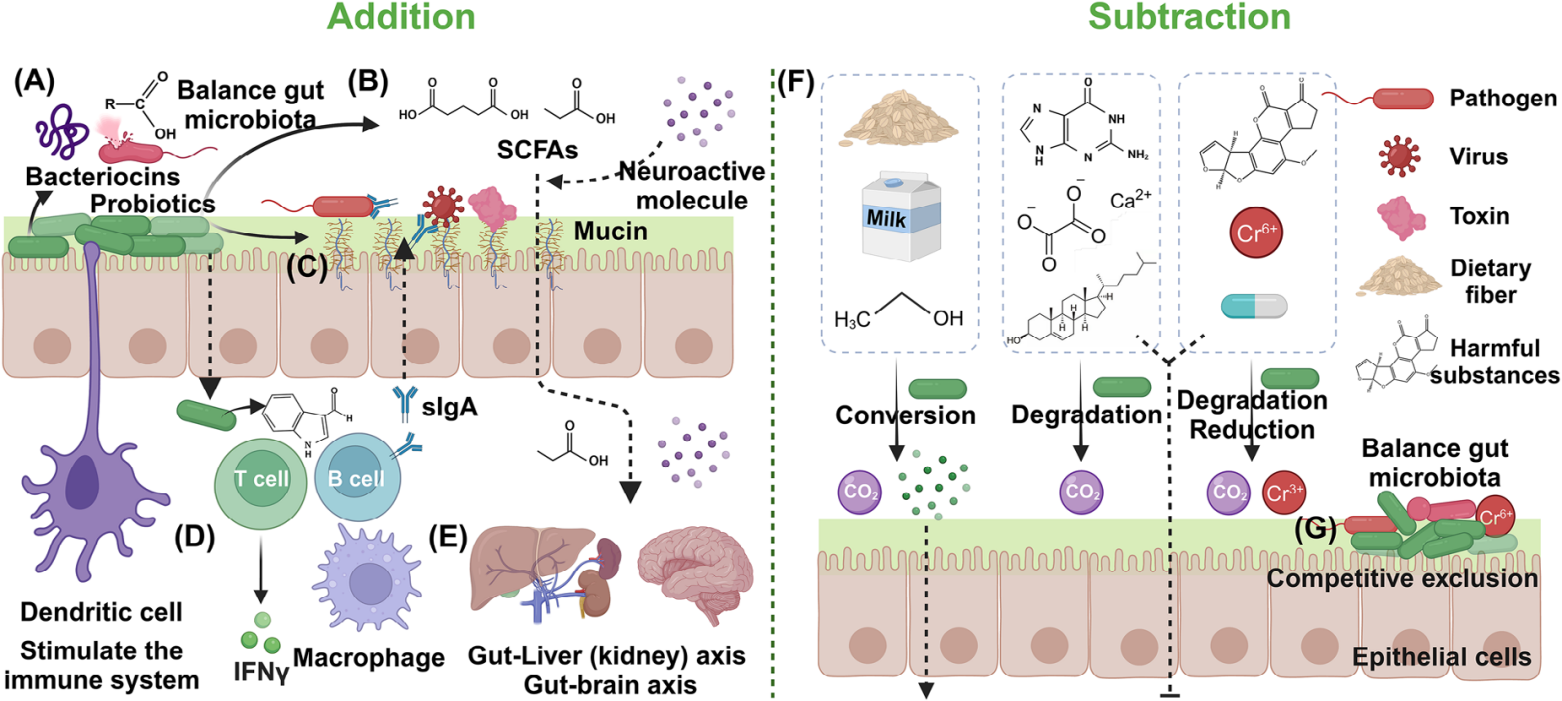
Primary therapeutic mechanisms of probiotics and postbiotics. (A) Production of antimicrobial substances. (B) Regulation of gut microbiome balance. (C) Increased adhesion to the intestinal mucosa and improvement of the epithelial barrier. (D) Stimulation of the immune system. (E) Affects the gut–brain axis, gut–kidney axis, and gut–liver axis. (F) Degradation or absorption the detrimental metabolites produced by metabolic diseases or harmful exogenous substances and metabolizes prebiotics to produce beneficial substances. (G) Competitive exclusion of pathogenic microorganisms.

Most human diseases are associated with dysbiosis in the gut microbiota, whose homeostasis plays a crucial role in maintaining health and delaying the development of various diseases.^75^ When the indigenous gut microbiota is disrupted, it can lead to an increased risk of diseases such as obesity, inflammation, and cancer.^155^ Probiotics, prebiotics, and postbiotics can improve the structure and function of gut microbiota, enhance intestinal barrier function, and promote the development and regulation of the immune system.^16^ Based on the current research on the role of probiotics and postbiotics, the mechanisms by which probiotics treats diseases mainly by affecting the gut microbiome, this process can be summarized as “addition” and “subtraction” mechanisms, and postbiotics play a very important role in the “addition” mechanism (Figure 4). The “addition” mechanism involves modulation of gut microbiota through postbiotics secreted by probiotics, thereby treating, or delaying disease progression. These postbiotics include SCFAs, proteins, peptides, hormones, and neuroactive compounds, which contribute to the overall health of the host.^17^, ^23^ The “subtraction” mechanism focuses on the metabolic capabilities of functional probiotic bacteria. By degrading or absorbing detrimental metabolites produced by metabolic diseases and harmful exogenous substances, these probiotic bacteria reduce the intestinal absorption of these harmful components and lessen their impact on the body.^156^ This process simultaneously shaping the gut microbiota and reducing shock, this process ultimately contributes to the alleviation and treatment of the corresponding diseases.^151^

#### 4.3.1 The “addition” mechanism of probiotics

Probiotics exhibit an “addition” mechanism in treating diseases through several key aspects. Probiotics work to restore and maintain a balanced gut microbiota by producing substances like postbiotics SCFAs.^157^ This process improves digestion, nutrient absorption, and overall gut health while preventing the overgrowth of pathogenic bacteria and reducing inflammation (Figures 4A and B). In the enhancement of intestinal mucosal barrier function, certain functional probiotics can enhance the intestinal mucosal barrier function by promoting the production of tight junction proteins and mucin. This helps prevent the infiltration of pathogens and toxins into the bloodstream (Figure 4C).^158^ Probiotics also play a crucial role in immunomodulation, interacting with the host's immune system to modulate both innate and adaptive immunity (Figures 4D and E).^159^ This interaction stimulates cytokine production, activates natural killer cells, and boosts the function of antigen‐presenting cells, resulting in a more balanced and effective immune response.^160^ Last, probiotics reduce the risk of infection by inhibiting the growth and colonization of pathogenic bacteria in the gut by producing antimicrobial substances such as rumenococcin C and organic acids.^161^, ^162^

#### 4.3.2 The “subtraction” mechanism of probiotics

The “subtraction” mechanism of probiotics in treating diseases encompasses several ways, such as breaking down harmful substances, detoxifying metabolic byproducts, reducing ammonia levels, degrading complex dietary components such as prebiotics, and competing with pathogens for resources. By metabolizing and degrading harmful exogenous substances like toxins, carcinogens, antibiotics and even reducing heavy metals, probiotics help in neutralizing adverse effects of harmful substances on the gut microbiota and the body (Figure 4F).^123^ Probiotics also aid in breaking down and eliminating unfavorable metabolites such as uric acid and acetate produced during metabolic processes, promoting a healthy gut environment, providing liver protection and even relieving gout caused by high uric acid.^126^, ^129^ Certain probiotics are capable of breaking down complex dietary components, including lactose, oxalates, and phytic acid, rendering them more digestible and minimizing the risk of intolerance or malabsorption‐related symptoms.^97^ Some dietary prebiotics will also be selectively metabolized by probiotics to SCFAs and other postbiotics to promote the growth of beneficial microorganisms while maintaining the health of the host.^23^ Additionally, probiotics compete with pathogens for adhesion sites and nutrients, restricting pathogen colonization and proliferation within the gut, and this mechanism is also very important for maintaining the balance of gut microbiota (Figure 4G).^163^ Leveraging the ability of functional probiotics to target and degrade harmful substances or prebiotics, the impact on gut microbiota and overall health is mitigated. Probiotics work together with the shaped gut microbiota to provide relief and treatment for associated disease symptoms. Li and coworkers^164^ also defined the mechanism of probiotics in treating diseases through a “subtraction” approach as “gut remediation” and have conducted extensive research in this area.

## 5 EFFECTIVENESS AND MECHANISMS OF PREBIOTICS IN TREATING DISEASES

### 5.1 Effectiveness of prebiotics in animal model therapy and clinical trials

Numerous animal studies have demonstrated that prebiotics can selectively stimulate the growth and activity of beneficial bacteria in the gut, thereby improving host health. A recent study by Szklany et al.^165^ found that supplementing the diet of adolescent rats with a prebiotic mixture (including GOS and FOS) reduced anxiety‐like behavior and altered the gut microbiota, demonstrating potential as a novel therapy for anxiety disorders. Fascinatingly, prebiotics may even influence sleep quality; the prebiotic GOS and polydextrose (PDX) could alleviate stress‐induced sleep disturbances in rats. The GOS/PDX prebiotic diet increased the relative abundance of *Parabacteroides distasonis* and altered the fecal bile acid pool, and these changes were associated with improved sleep patterns in stressed rats, indicating a potential role for prebiotics in treating stress‐related sleep disorders.^166^ In the field of metabolic diseases, prebiotic xylooligosaccharides (XOS) could mitigate high‐fat diet (HFD)‐induced metabolic syndrome in rats by increasing the number of *Bifidobacteria* and *Lactobacilli* and reducing the abundance of *Proteobacteria*.^167^ Nonalcoholic fatty liver disease (NAFLD) is the most common metabolic disease worldwide, and it may also reduce the insulin resistance in NAFLD patients through a chain reaction to induce hyperinsulinemia and increase the risk of bladder cancer.^168^ Doctors are currently considering natural products to alleviate NAFLD, and prebiotics are one of the options.^169^ FOS and inulin reduce de novo fat synthesis in rats fed a HFD by regulating the expression of lipogenic enzyme genes and reducing fatty acid synthase activity, while attenuating liver weight and steatosis.^170^, ^171^ Furthermore, they restore intestinal permeability and reverse HFD‐induced gut microbiota dysbiosis. Prebiotic GOS and polyphenols supplementation can also reduce liver fat accumulation, decrease systemic inflammation, and improve insulin sensitivity in mice.^172^ The changes in gut microbiota were correlated with improved glucose homeostasis and lipid metabolism, demonstrating the potential of prebiotics in managing metabolic diseases.^173^ Similarly, the use of XOS to improve gut health and protect against gastrointestinal diseases is being investigated. Fei et al.^174^ demonstrated that XOS ameliorated symptoms of colitis in mice, partly through promoting SCFAs production by increasing the abundance of SCFA‐producing bacteria *Prevotella* and *Paraprevotella*. These effects were associated with an increase in beneficial bacteria such as *Lactobacillus* and *Bifidobacteria*, and increased production of SCFAs.^172^, ^175^ The mechanism by which probiotic feeding selectively increases the abundance of probiotics to treat diseases also illustrates the importance of probiotics in disease treatment.

The potential of prebiotics to modulate gut microbiota and improve health outcomes has not been limited to animal models but also observed in recent clinical trials. In an open‐label, nonrandomized study involving patients with PD, Hall et al. demonstrated that inulin supplementation can promote the growth of genera *Blautia*, *Anaerostipes*, and *Bifidobacterium* and increase intestinal SCFAs production in PD patients, improving the gut barrier, reducing inflammation and lowers levels of the neurodegenerative disease marker NfL (NCT04512599).^176^ The effectiveness of prebiotics in metabolic diseases such as obesity and diabetes has also been verified. Oligofructose‐enriched inulin selectively alters the gut microbiota and significantly reduces weight z‐scores, percent body fat in overweight or obese children (NCT02125955).^177^ Oligofructose‐enriched chicory also has beneficial effects on improving blood glucose and calcium homeostasis, liver function tests, blood pressure, and reducing hematological risk factors for diabetes in female patients with type 2 diabetes.^178^ Prebiotics also have a beneficial effect on gastrointestinal diseases. Silk et al.^179^ studied the efficacy of prebiotic trans‐GOS in changing the colonic microbiota and improving the symptoms of IBS patients, and found that GOS specifically stimulated gut *Bifidobacteria* in IBS patients and effectively relieved IBD symptoms (Registered in ISRCTN, ISRCTN54052375). A synbiotic formulation containing *Lactobacillus*, *Bifidobacterium* probiotic strains, and short‐chain FOS improves associated symptoms such as flatulence and bowel habits in patients with diarrhea‐predominant IBS in a randomized double‐blind, placebo‐controlled study (NCT04206410).^180^ These findings suggest that prebiotics have potential as therapeutic agents in a variety of diseases.

### 5.2 Mechanisms of prebiotics in maintaining health and treating diseases

**FIGURE 5:**
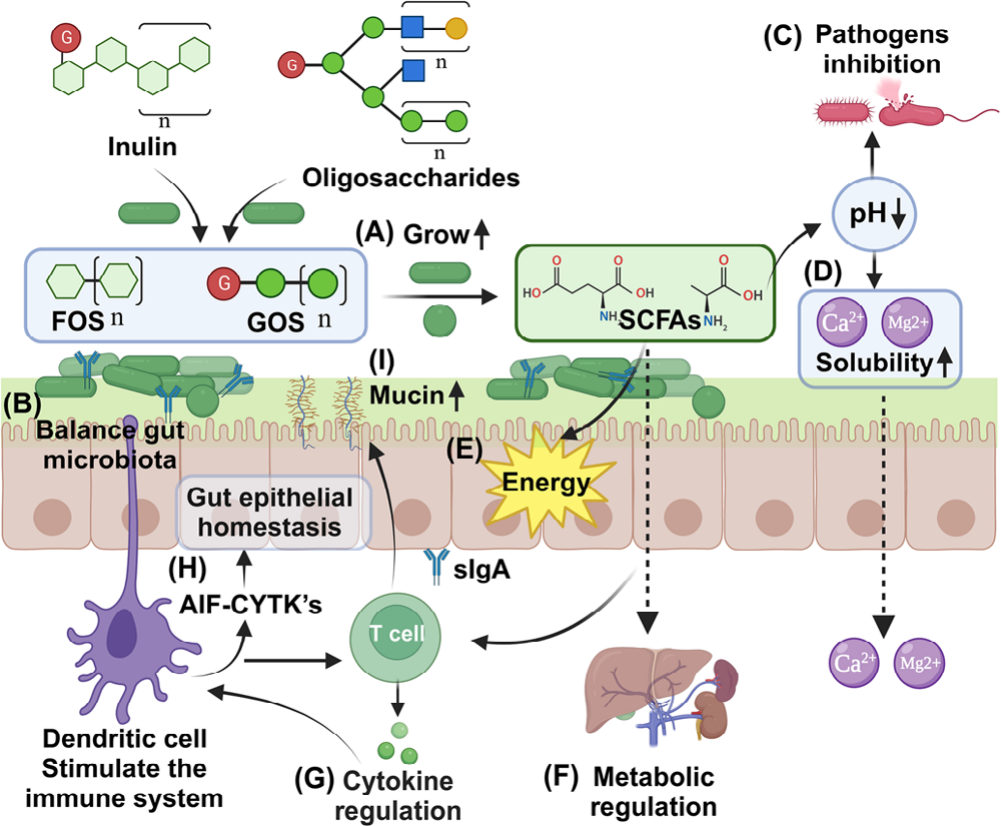
The main mechanisms of prebiotics to maintain health and treat diseases. (A) Promotes the growth of beneficial microorganisms, (B) balances the gut microbiota. The produced SCFAs lower the pH of the intestinal lumen, (C) avoid pathogen colonization, and (D) increase the dissolution and absorption of Ca^+2^ and Mg^2+^. (E) SCFAs provide an energy source for intestinal epithelial cells, (F) regulate fat metabolism, and (G) promote cytokine production. (H) Stimulates anti‐inflammatory cytokines (AIF‐CYTK's) production to maintain intestinal epithelial homeostasis, and (I) induces mucin production in enterocytes. FOS, fructooligosaccharides; GOS, galactooligosaccharides.

The primary role of prebiotics is to serve as substrates for the fermentation of specific beneficial gut microbes. By offering these microbes a competitive growth advantage, prebiotics can influence the overall composition of the gut microbiota, enhancing its diversity.^181^ Beneficial bacteria associated with health benefits, such as *Bifidobacteria* and *Lactobacilli*, proliferate abundantly under the influence of prebiotics like FOS and GOS (Figure 5A). This preferential growth inhibits the colonization and multiplication of potential pathogens, helping to foster a healthier gut environment and maintains a balanced gut microbiota (Figure 5B).^182^, ^183^ When gut microbes ferment prebiotics, they produce SCFAs, including acetate, propionate, and butyrate.^184^ These SCFAs play diverse roles in maintaining gut health, helping to regulate luminal pH, creating an environment that suppresses pathogen growth while favoring beneficial microbes (Figure 5C).^185^ Furthermore, a lower pH enhances the solubility of mineral salts, improving mucosal function and absorption surfaces (Figure 5D). This boosts the absorption of essential minerals like calcium and magnesium, playing a crucial role in osteoporosis prevention.^186^ Notably, butyrate among SCFAs is also serves as the primary energy source for colonic cells, promoting a healthy gut barrier with anti‐inflammatory properties (Figure 5E).^187^ SCFAs enter the portal stream and reach liver tissue where they can promote fatty acid oxidation in the liver (Figure 5F).^184^

Prebiotics can impact immune functions in various ways. By stimulating beneficial bacteria, they can affect the gut‐associated lymphoid tissue, promoting the production of cytokines and chemokines (Figure 5G).^188^ For instance, inulin‐type fructans can stimulate dendritic cells, leading to the production of anti‐inflammatory cytokines (AIF‐CYTK's), maintain intestinal epithelial homeostasis and offering potential therapeutic pathways for IBD (Figure 5H).^189^ A robust gut barrier prevents the translocation of pathogens and their toxins into the systemic circulation. Several cytokines can stimulate intestinal cells to produce mucin, reinforcing the gut barrier and reducing risks associated with infections and systemic inflammatory responses (Figure 5I).^190^, ^191^ Prebiotic therapy stands at the crossroads of nutrition, microbiology, and medicine. Its mechanisms, from direct gut microbiota modulation to broader metabolic and immune effects, underscore its potential as a tool for prevention and treatment. As our understanding of these interactions deepens, prebiotic therapies are likely to play an increasingly indispensable role in health maintenance and disease treatment in the coming years.

## 6 CHALLENGES OF PROBIOTICS, PREBIOTICS, AND POSTBIOTICS AS MEDICINE IN CLINICAL APPLICATION

Although probiotics, prebiotics, and postbiotics have achieved certain breakthroughs in clinical applications, they also face several challenges and limitations. Addressing these limitations is necessary to realize their full therapeutic potential and to be accepted by more people.^192^, ^193^ Probiotics strain specificity plays a crucial role, as the effectiveness of probiotics can vary significantly depending on the bacterial strains.^194^ Identifying and selecting the most appropriate strain for a specific condition requires further research and evaluation. The lack of standardization in terms of probiotic strain, dose, and prebiotics formulation poses challenges in comparing study results and determining the best therapeutic approach.^195^ Quality control is vital for the effectiveness of probiotic products; however, inconsistencies in manufacturing processes and storage conditions can affect product quality, leading to variations in clinical outcomes.^196^ In terms of the above challenges, prebiotics have more advantages than probiotics, but they still need the assistance of probiotics to exert their functions.^197^ Regulatory concerns arise due to the varying classification of probiotics, prebiotics, postbiotics as dietary supplements, food additives, or medicines among countries, leading to differing regulatory standards and approval processes.^198^ The consumption of live bacteria in probiotics or certain types of prebiotics and postbiotics may conflict with some cultural or religious beliefs. This can create challenges in terms of product development, registration, and market access. As probiotics sit at the intersection of medicine, nutrition, and even ecological science, ethical best practices from multiple disciplines must be considered.

The safety of probiotics, prebiotics, and postbiotics are an important consideration, especially in immunocompromised individuals, critically ill patients, children, and those with central venous catheters, as they may be at an increased risk of infections or complications.^199^, ^200^, ^201^ Interactions between probiotics or prebiotics and other medications can impact treatment outcomes, making it essential to identify and manage potential interactions in clinical practice.^202^ Patient compliance can be challenging due to factors such as taste, convenience, and cost. Individual variability due to differences in gut microbiota, genetics, diet, and other factors necessitates a personalized therapy, requiring more extensive research and understanding.^203^ Through short‐term probiotic interventions, changes in the gut microbiota might be temporary, and some short‐term probiotic treatment trials have failed to detect significant alterations in the gut microbiota, and special individuals or diseases may require a longer treatment duration to achieve therapeutic effects.^204^ Additionally, the vast majority of studies on the impact of probiotics and prebiotics on health through the gut–brain axis have been conducted on animal models, and there are still gaps in our understanding of their interactions with the human body and mechanisms for treating diseases. Limited clinical evidence and inconclusive or contradictory findings, partly due to limitations in study design, sample size, and duration, warrant more rigorous and well‐designed trials.^198^ Some of the precise mechanisms through which probiotics, prebiotics, and postbiotics exert their therapeutic effects are not yet fully understood, making it challenging to optimize their use for specific conditions.^205^ Addressing these challenges and limitations will be essential for the successful clinical application of probiotics, prebiotics, and postbiotics, including conducting more extensive research, optimizing strain selection, ensuring quality control, and regulatory compliance. Developing personalized probiotic‐related therapies and identifying potential medication interactions will help enhance treatment outcomes and reduce potential risks for patients.

## 7 FUTURE PERSPECTIVES

The probiotics related market is experiencing significant growth due to increased consumer awareness of gut health and scientific advancements in microbiome research. According to a GLOBE NEWSWIRE report titled “Global Probiotics Industry,” the global probiotics market is expected to reach USD 91.7 billion by 2030 (https://www.reportlinker.com/p05336745/?utm_source=GNW). This accelerates the development of innovative probiotic formulations and personalized treatments based on individual gut profiles.^206^ In the field of prebiotics, according to a report by Grand View Research, the global prebiotics market size in 2021 is USD 6.05 billion. Rising use of prebiotics in the dairy industry and increasing technological advancements in developing inulin and oligosaccharides will also drive the market, and the market size is expected to grow at a compound annual growth rate of 14.9% from 2022 to 2030 (https://www.grandviewresearch.com/industry‐analysis/prebiotics‐market). With expanding applications in areas such as mental health and metabolic diseases, the market potential for probiotics related is broadening. Regulatory changes and collaboration between academia, biotech, and pharmaceutical sectors further support the development of these therapies.^207^, ^208^ Overall, market trends and prospects for probiotics, prebiotics, and postbiotics appear promising, with continued growth expected as targeted therapies and consumer demand increase.

Probiotics, prebiotics, and postbiotics have the potential to revolutionize public health and the healthcare system by providing targeted and personalized treatments for various health conditions.^203^ By harnessing the functions of probiotic bacteria and prebiotics, maintain a healthy gut microbiota, strengthen the immune system, and address a range of issues from gastrointestinal disorders to mental health.^70^ The widespread adoption of probiotic therapies could lead to improved patient outcomes, reduced reliance on antibiotics, and lowered healthcare costs.^209^ Furthermore, integrating probiotics related products into preventative healthcare measures could promote overall well‐being and minimize the burden of chronic diseases on the healthcare system.

The current development direction of probiotics, prebiotics, and postbiotics focuses on personalized and targeted therapies, aiming to harness the full potential of the gut microbiome for various health conditions.^210^ Advancements in next‐generation sequencing, metagenomics, and bioinformatics are enabling researchers to better understand the complex interactions between gut microbiota and host health, facilitating the development of innovative treatments.^211^ Emerging technologies such as CRISPR‐based genetic editing and high‐throughput screening methods are being used to identify and modify specific probiotic strains for enhanced therapeutic potential.^212^, ^213^ If engineered probiotics are approved in the medical field, the clinical application and market development of probiotics may be further accelerated. Additionally, the integration of artificial intelligence and machine learning in medicine discovery is accelerating the identification of novel probiotics, prebiotics, and postbiotics candidates and optimization of their formulations. Research on microencapsulation and delivery systems is improving the stability, viability, and targeted delivery of probiotics, enhancing their efficacy in clinical applications.^214^ These emerging technologies are advancing probiotic‐related R&D, paving the way for more effective and tailored interventions in the prevention and treatment of various health conditions.

Probiotics, prebiotics, and postbiotics hold great potential as next‐generation therapies, offering personalized and targeted treatments for various health conditions. Their effectiveness is attributed to their ability to modulate the gut microbiota, improving overall health, and addressing specific diseases. However, some mechanisms of action of probiotics, prebiotics, and postbiotics; the safety and efficacy of probiotics and prebiotics, probiotic synthetic microbiota, and even synthetically engineered probiotics in clinical applications; the clinical dosage of prebiotics and activity of probiotics; and ensuring patient, public, and regulatory acceptance of probiotic‐related therapies remain challenging. Resolving these issues will be crucial for the successful integration of probiotics, prebiotics, and postbiotics into mainstream medicine. In addition, given the effectiveness and unique advantages of probiotics and their metabolites in treating a variety of diseases, and considering the definitions of medicines and “live biotherapeutics,”^62^ we hope to propose the term “probacine” (PRObiotic BActerial mediCINE) to emphasize the role of probiotics in the prevention, alleviation, and treating diseases, and further promote the clinical application of probiotics, prebiotics, and postbiotics.

## 8 CONCLUSIONS

Extensive research and clinical evidence have demonstrated the mechanisms and effectiveness of probiotics, prebiotics, and postbiotics in restoring gut microbiota homeostasis and treating a variety of diseases. Probiotics can alleviate or treat diseases by regulating the gut microbiota by producing postbiotics through “addition,” by removing harmful metabolites and exogenous substances through “subtraction” to reduce their impact on the body. Prebiotics selectively stimulate beneficial microorganisms, improve gut microbiota to maintain health and treat disease. In recent years, the development of research technologies, such as sequencing and artificial intelligence, along with the in‐depth study of the mechanisms of probiotics, prebiotics, and postbiotics, will promote the development of probiotics‐related fields and address current challenges. Overall, the field of probiotics research is full of promise and excitement. Based on current research and clinical evidence, probiotics, prebiotics, and postbiotics will play an even more important role in clinical treatments in the future and may become the next generation of representative medicines that will revolutionize the way we treat and manage diseases.

## AUTHOR CONTRIBUTION

Shuang‐Jiang Liu, Zuoyi Jiao, and Xiangkai Li conceived of the paper. Jing Ji and Weilin Jin wrote and edited the paper. Weilin Jin and Xiangkai Li revised the paper. All authors have read and approved the final paper.

## CONFLICT OF INTEREST STATEMENT

The authors declare that there are no conflict of interest.

## ETHICS STATEMENT

Not applicable.

## Data Availability

Not applicable.
